# Characterization of a Novel Anti-Cancer Compound for Astrocytomas

**DOI:** 10.1371/journal.pone.0108166

**Published:** 2014-09-25

**Authors:** Sang Y. Lee, Becky Slagle-Webb, Elias Rizk, Akshal Patel, Patti A. Miller, Shen-Shu Sung, James R. Connor

**Affiliations:** 1 Department of Neurosurgery, Pennsylvania State University College of Medicine, Penn State M.S. Hershey Medical Center, Hershey, Pennsylvania, United States of America; 2 Department of Radiology, Pennsylvania State University College of Medicine, Penn State M.S. Hershey Medical Center, Hershey, Pennsylvania, United States of America; 3 Department of Pharmacology, Pennsylvania State University College of Medicine, Penn State M.S. Hershey Medical Center, Hershey, Pennsylvania, United States of America; University of Navarra, Spain

## Abstract

The standard chemotherapy for brain tumors is temozolomide (TMZ), however, as many as 50% of brain tumors are reportedly TMZ resistant leaving patients without a chemotherapeutic option. We performed serial screening of TMZ resistant astrocytoma cell lines, and identified compounds that are cytotoxic to these cells. The most cytotoxic compound was an analog of thiobarbituric acid that we refer to as CC-I. There is a dose-dependent cytotoxic effect of CC-I in TMZ resistant astrocytoma cells. Cell death appears to occur via apoptosis. Following CC-I exposure, there was an increase in astrocytoma cells in the S and G2/M phases. In *in vivo* athymic (*nu*/*nu*) nude mice subcutaneous and intracranial tumor models, CC-I completely inhibited tumor growth without liver or kidney toxicity. Molecular modeling and enzyme activity assays indicate that CC-I selectively inhibits topoisomerase IIα similar to other drugs in its class, but its cytotoxic effects on astrocytoma cells are stronger than these compounds. The cytotoxic effect of CC-I is stronger in cells expressing unmethylated O^6^-methylguanine methyltransferase (MGMT) but is still toxic to cells with methylated MGMT. CC-I can also enhance the toxic effect of TMZ on astrocytoma when the two compounds are combined. In conclusion, we have identified a compound that is effective against astrocytomas including TMZ resistant astrocytomas in both cell culture and *in vivo* brain tumor models. The enhanced cytotoxicity of CC-I and the safety profile of this family of drugs could provide an interesting tool for broader evaluation against brain tumors.

## Introduction

Gliomas account for 28% of all primary brain and central nervous system (CNS) tumors, and 80% of gliomas are malignant [1]. Among gliomas, glioblastoma (glioblastoma multiforme, grade IV astrocytoma, GBM) is the most common malignant glioma. The mortality rate of primary malignant brain and CNS tumors is high; approximately 22,620 new adult cases of malignant brain and CNS cancers in 2013 [1] and 13,700 deaths occurred in 2012 [2]. The median survival for GBM patients was 14.6 months and the 2 year survival of patients with GBM was 10.4% for radiotherapy alone and only 26.5% undergoing combined therapy treatment of temozolomide (TMZ) and radiation [3].

The current standard treatment for GBM is total resection followed by radiotherapy alone or combination with TMZ chemotherapy [4], [5]. TMZ is an oral alkylating agent used in the treatment of brain cancer, *e.g.*, GBM and oligodendroglioma [6]. It has also been used to treat melanoma, prostate cancer, pancreatic carcinoma, soft tissue sarcoma, and renal cell carcinoma [7]–[11]. TMZ inhibits cell reproduction by inhibiting DNA replication [12] and has unique characteristics compared with other alkylating agents. For example, it is administered orally, crosses the blood-brain barrier, is less toxic than other alkylating agents, and does not chemically cross-link DNA. However, although TMZ is the current chemotherapeutic standard for treating brain tumors and other cancers, as many as 50% of brain tumors are resistant to TMZ therapy [13], [14]. In addition, almost all tumors eventually come back and the large majority of recurrent tumors are resistant to chemotherapy [15], [16]. Therefore, the development of new treatment options including novel drugs for therapy resistant brain tumors is urgently needed.

In addition to the alkylation agents like TMZ, topoisomerase inhibitors are another group of anti-cancer drugs under evaluation. Topoisomerases are important nuclear enzymes that regulate the topology of DNA, maintain genomic integrity and are essential for DNA replication, recombination, transcription and chromosome segregation [17]. There are six human topoisomerase enzymes [18] and three of them, topoisomerase I, topoisomerase IIα and topoisomerase IIβ, have significant involvement in cancer and cancer chemotherapy [19]. The topoisomerase I enzyme nicks and rejoins one strand of the duplex DNA, and topoisomerase II enzyme transiently breaks and closes double-stranded DNA [20]. The topoisomerase I inhibitors (e.g., topotecan) have been used in patients with recurrent small-cell lung cancer, recurrent malignant gliomas, recurrent childhood brain tumors [21], [22]. Although topoisomerase II inhibitors were studied in glioma cells [23]–[25], the topoisomerase II inhibitors haven't been widely used in adults with primary brain tumors due to their poor CNS penetrance. Therefore, small molecules with the capability to penetrate the brain would be highly desirable to treat gliomas *in vivo*.

We have previously reported that human neuroblastoma cells and human astrocytoma cells lines expressing commonly occurring polymorphisms in the HFE gene were resistant to chemotherapy and radiation [26]. The HFE gene product is involved in iron homeostasis and the common HFE polymorphisms, H63D and C282Y, lead to a number of changes in cells such as increased endoplasmic reticulum stress and increased oxidative stress [27]–[29]. In the present study, we used astrocytoma cell lines that we identified with the HFE gene variants and TMZ resistance to screen compounds from DIVERSet compound library from Chembridge (San Diego, CA) and found a number of effective compounds with a similar chemotype. We identified an analog of a thiobarbituric acid compound which has strong toxic effect on TMZ-resistant astrocytoma cells. We report here the characterization of the lead compound in *in vitro* cell culture and *in vivo* brain tumor models.

## Materials and Methods

### Materials

Dulbecco’s Modified Eagle Medium (DMEM), fetal bovine serum (FBS) and other cell culture ingredients were purchased from Life Technologies (Grand Island, NY). All the PCR Array ingredients were supplied from SABiosciences (Frederick, MD). TMZ was purchased from Oakwood Products Inc. (West Columbia, SC) and was dissolved in cell culture medium or 100% DMSO. The lead chemotype compound–I (CC-I) was ordered from ChemBridge Corporation (San Diego, CA). The compound was dissolved in DMSO as a stock solution and diluted for the experiment. Topoisomerase enzymes I and IIα assay kits were ordered from TopoGen Inc. (Port Orange, FL). Merbarone was obtained from Calbiochem (San Diego, CA). All of the other chemicals used were purchased from Sigma Co. (St. Louis, MO).

### Human astrocytoma cell culture, treatment and cytotoxicity assay

Human astrocytoma cells (SW1088-grade III, U87-MG-grade IV, CCF-STTG1-grade IV, T98G-grade IV, LN-18-grade IV) were ordered from American Type Culture Collection (ATCC, Manassas, VA) and maintained in DMEM (Gibco by Life Technologies, catalog 11885) supplemented with 100 U/mL penicillin, 100 µg/mL streptomycin, 0.29 mg/mL L-glutamine, and 10% FBS. All experiments were performed at 37°C in 5% CO_2_ atmosphere cell culture conditions. For the cytotoxicity assays, the compounds tested were prepared by first diluting them from the stock solution in cell culture media. The compounds were exposed to the cells for 3–6 days. Cell cytotoxicity was performed by MTS [3-(4,5-dimethylthiazol-2-yl)-5-(3-carboxymethoxyphenyl)-2-(4-sulfophenyl)-2H-tetrazolium] cell proliferation assay (Promega, Madison, WI) or sulforhodamine B (SRB) assay at the end of the cell culture period.

### Acute toxicity determination

Acute toxicity of CC-I was determined in athymic nude mice (strain 088 or 490, Charles River Laboratories, Wilmington, MA) according to the NIH drug development program’s acute toxicity procedure with minor modification. To determine the acute toxicity, a total of six female mice (1–2 month old) were injected intraperitoneally with 3 different doses (e.g., 20 mg/kg, 37.5 mg/kg, 50 mg/kg) of CC-I or vehicle control once a week and then observed for a period of 7–14 days. The mice were observed daily for changes in body weight, visible and/or palpable dermal infection, presence of ascites, food consumption or nutrition status, and grooming or impaired mobility or death to determine acute toxicity. At 7–14 days after treatment, 0.5–1 ml of blood was collected through a cardiac heart puncture while the mice were under anesthesia (Ketamine 100 mg/kg body weight/xylazine 10 mg/kg body weight, intraperitoneally) for blood toxicity examination. All the animals in the study were housed in germ-free environmental rooms, and individual bubble systems. All the animal experiments were approved (IACUC #2011-062) by the Pennsylvania State University Institutional Animal Care and Use Committees.

### Subcutaneous tumor model

To test the anti-tumor effect of CC-I against human astrocytoma tumor, one-two month old female immunodeficient (*nu*/*nu*) nude mice (strain 088, Charles River Laboratories, Wilmington, MA) were implanted 10×10^6^ cells per mouse subcutaneously with TMZ sensitive SW1088 or TMZ resistant CCF-STTG1 astrocytoma cells. When the tumor reached approximately 32–100 mm^3^ in size, the mice (n = 10 or 11) were randomly divided into two groups. The CC-I was injected intraperitoneally at a concentration of 25 mg/kg body weight in a volume of 200–300 µL in 12.5% ethanol once a week for 7 weeks. The control group was given phosphate-buffered saline (PBS) in the same volume and regimen. Tumor size was measured weekly with a Vernier caliper for 7 weeks by an investigator blinded to experimental conditions. Tumor volume (V) was calculated according to the formula V = *a* ^2^/2×b, where *a* and *b* are minor and major axes of the tumor foci, respectively. The tumor size, health, and survival of the mice were visibly monitored daily and the tumor size measured weekly. We did not take pictures of the tumors. We will consider taking pictures for upcoming experiments. To monitor the toxicity of compounds, the animals were euthanized with ketamine/xylazine 100/10 mg/kg body weight intraperitoneally, and measured liver and kidney toxicity at the end of the experiment.

### Intracranial xenograft model

Female immunodeficient nude mice (strain 088, Charles River Laboratories, Wilmington, MA) weighing 20–30 g were anesthetized by intraperitoneal injection of ketamine-xylazine 100 mg/kg–10 mg/kg body weight. Human U87-MG and CCF-STTG1 astrocytoma cell lines were implanted to create the brain tumor xenograft. In brief, the head was held in horizontal position and 1 million astrocytoma cells in a volume of 10 µL were injected slowly into the caudate putamen region using a small animal stereotactic apparatus. The stereotactic co-ordinates used for the xenografts are P = 0.5, L = 1.7, H = 3.8 mm. The astrocytoma cells were injected slowly for 10 minutes to avoid elevation in the intracranial pressure or upward cell suspension leakage through the track of the needle. The animals were given buprenorphine (0.05–0.1 mg/kg body weight subcutaneous) for pain during and after surgery. This was given every 8–12 hours for 24–48 hours after surgery. The animals were subjected to T1 weighted magnetic resonance imaging (MRI) twice; once to determine that a tumor is established in the brain (∼3 weeks injection of astrocytoma cells) and at the end of the experiment. The animals were monitored on a daily basis and the body weight was recorded weekly. Once a tumor was observed, the mice (n = 12 or 15) were randomly divided into two groups. CC-I (25 mg/kg body weight) or PBS was injected once a week intraperitoneally. The overall survival of mice was performed by a Kaplan-Meier survival curve. The animals were euthanized according to acceptable method of euthanasia as defined by the American Veterinary Medical Association (AVMA) Guidelines on Euthanasia - Approved Euthanasia Methods, 2013. Once the animals receive a body condition score of less than 2, the animals were euthanized with ketamine/xylazine 100/10 mg/kg body weight intraperitoneally as well as a secondary method of cervical dislocation. At the termination of the experiment, plasma was collected for analysis of liver and kidney toxicity after euthanized with ketamine/xylazine 100/10 mg/kg body weight intraperitoneally.

### T1 weighed MRI images

T1 weighted MRI contrast was used to visualize the tumor growth using 7T MRI system (Bruker, Biospec GmbH, Ettlingen, Germany). The imaging parameters of the T1 scan are TR/TE = 540 ms/11 ms, 8 averages, 192×192, 0.5 mm slice thickness, and 3.2 cm^2^ FOV. The mice were anesthetized by inhalation of 1–2% isoflurane and placed in a position with brain located at the center of the coil. Intracranial tumor volume was estimated using Gadolinium enhanced T1 weighed multislice axial fast spin echo images. From these images the size of the tumor was calculated using the Region-of-Interest tool available on the Paravision software (Bruker Biospec, Ettlingen, Germany).

### Liver and kidney toxicity

The liver and kidney toxicity (total bilirubin, blood urea nitrogen (BUN), creatine, aspartate aminotransferase (AST), alanine aminotransferase (ALT), and alkaline phosphatase) was assessed for both subcutaneous tumor model and intracranial xenograft model using an automated chemistry analyzer (Roche Cobase MIRA) and kits manufactured by Thermo Electron (Louisville, CO). The blood was obtained from the control or CC-I injected mice with astrocytoma cells at the termination of the experiment.

### Apoptosis assay

For apoptosis assay, the 3×10^6^ of CCF-STTG1 cells were cultured for 48 hr with several concentrations (∼36 µM) of CC-I or actinomycin D (∼80 nM) as a positive control. The cells were harvested following trypsine-EDTA exposure and washed in cold PBS. Then 100 µL of the cell suspension (∼1×10^6^ cells) was incubated with 1 µL of 100 µg/mL red-fluorescent propidium iodide nucleic acid binding dye and 5 µL Annexin V-FITC (Molecular Probes, Carlsbad, CA) for 15 minutes at room temperature in the dark. The cells were analyzed by flow cytometry (Becton Dickinson, Franklin Lakes, NJ) of emission at 530 nm (e.g. FL1) and >575 nm (e.g. FL3). The cells that are bound by Annexin V illustrate early apoptotic cells. Cells that are reactive for both Annexin V and propidium iodide are necrotic cells.

### Gene expression profiling

We used Apoptosis PCR Array (SABiosciences, Frederick, MD) to determine which genes are altered by CC-I in TMZ resistant CCF-STTG1 cells. The PCR Array was performed according to the manufacturer’s instructions. In brief, total RNA was extracted from vehicle (0.1% DMSO) treated or CC-I treated CCF-STTG1 cell lines using qPCR-Grade RNA Isolation kit. One µg of RNA was used for first strand cDNA synthesis by reverse transcription with MMLV reverse transcriptase. Then real-time PCR was performed with diluted cDNA and master mix with ROX filter. For signal detection, the ABI Prism 7900 Sequence Detector System was programmed with an initial sterilization step of 2 minutes at 50°C, followed by 10 minutes denaturation at 95°C and then 40 cycles for 15 second at 95°C, 1 minute at 60°C and 30 second at 72°C. Each reaction sample was performed in triplicate. PCR Array data was calculated by the ΔΔcycle threshold (ΔΔ*Ct*) method, then normalized against multiple housekeeping genes and expressed as mean fold changes in CC-I treated samples relative to vehicle treated control samples.

### Cell cycle analysis

For cell cycle analysis, CCF-STTG1 cells were cultured overnight at a density of 2–5×10^6^ cells per flask. The following day, the cells were treated with different concentrations of CC-I in fresh cell culture medium. After 24–48 hr later, the adherent cells were harvested and split (1×10^6^ cells per tube) for washing with HANK’s buffer, then fixed in ice-cold 70% ethanol overnight at −20°C. For DNA staining day, the cells were incubated with propidium iodide (100 µg/ml) and RNase A (20 µg/ml) for 15 min at 4°C (protect from light). Samples were analyzed using BD FACS Calibur Flow Cytometry Analyzer.

### Topoisomerase relaxation and decatenation assay

DNA relaxation and kinetoplast DNA (kDNA) decatenation assay was performed using topoisomerase I or II drug screening kit or Topopoisomerase II assay kit (TopoGEN, Inc., Port Orange, FL) according to the manufacturer's instructions [30]. Topoisomerase IIα decatenates kDNA which consists of highly catenated networks of circular DNA in an ATP-dependent reaction to yield individual minicircles of DNA. In brief, for topoisomerase IIα mediated kDNA decatenation assay, the 20 µL reaction mixture contains following components; 50 mM Tris-HCl, pH 8.0, 150 mM NaCl, 10 mM MgCl_2_, 0.5 mM dithiothreitol, 30 µg/mL bovine serum albumin, 2 mM ATP, 260 ng of kDNA, several concentrations of compounds, and 4 U of human topoisomerase IIα. The final concentration of 0.5% (v/v) DMSO was used because this concentration does not affect activity of topoisomerase IIα. The incubation of assay mixture was carried out at 37°C for 30 minutes and terminated by the addition of 4 µL stop loading dye. The kDNA decatenation products from the reaction mixture was resolved on a 1% agarose gel at 100 V for 40 minutes, then stained with 0.5 µg/mL ethidium bromide in TAE buffer (4 mM Tris base/glacial acetic acid [0.11% (v/v)]/2 mM Na_2_EDTA).

### Molecular modeling study

The molecular modeling studies were based on the X-ray crystal structure of human topoisomerase IIα bound to L-peptide at 1.50 Å resolution (PDB identification code: 2q5a) [31]. The position of the L-peptide was used to specify the dimensions of the CC-I binding site for the docking study. Docking between topoisomerase IIα protein and CC-I was carried out using the GLIDE program (Grid Based Ligand Docking from Energetics, from Schrödinger, L.L.C.) [32], [33]. The Jorgensen OPLS-2005 force field was employed in the GLIDE program. The optimal binding geometry for each model was obtained with GLIDE, which relies upon Monte Carlo sampling techniques coupled with energy minimization. GLIDE SP (Standard Precision mode) was used to dock the compound CC-I followed by GLIDE XP (Extra Precision mode). Schrödinger’s LigPrep was used to generate the 3D conformations of CC-I

### Statistical Analysis

All of the data was subjected to statistical analysis by the student *t*-test when comparing two groups. We used one-way ANOVA followed by Tukey-Kramer test for more than two group comparisons to determine if the differences are significant. For comparisons of time course or concentration data we performed repeated measures two-way ANOVA followed by Tukey-Kramer test. Differences among means are considered statistically significant when the *p* value is less than 0.05. The LC_50_ (50% lethal concentration) of compounds was determined using statistical software (GraphPad Prism 6) as a general indicator of a chemical's toxicity. In the *in vivo* brain tumor model, the tumor volume data was summarized as the mean values with standard errors. The mice survival was compared between the groups using Kaplan-Meier survival analysis with logrank test.

## Results

### Identification of a cytotoxic compound against TMZ resistant astrocytoma cells

**Figure 1:**
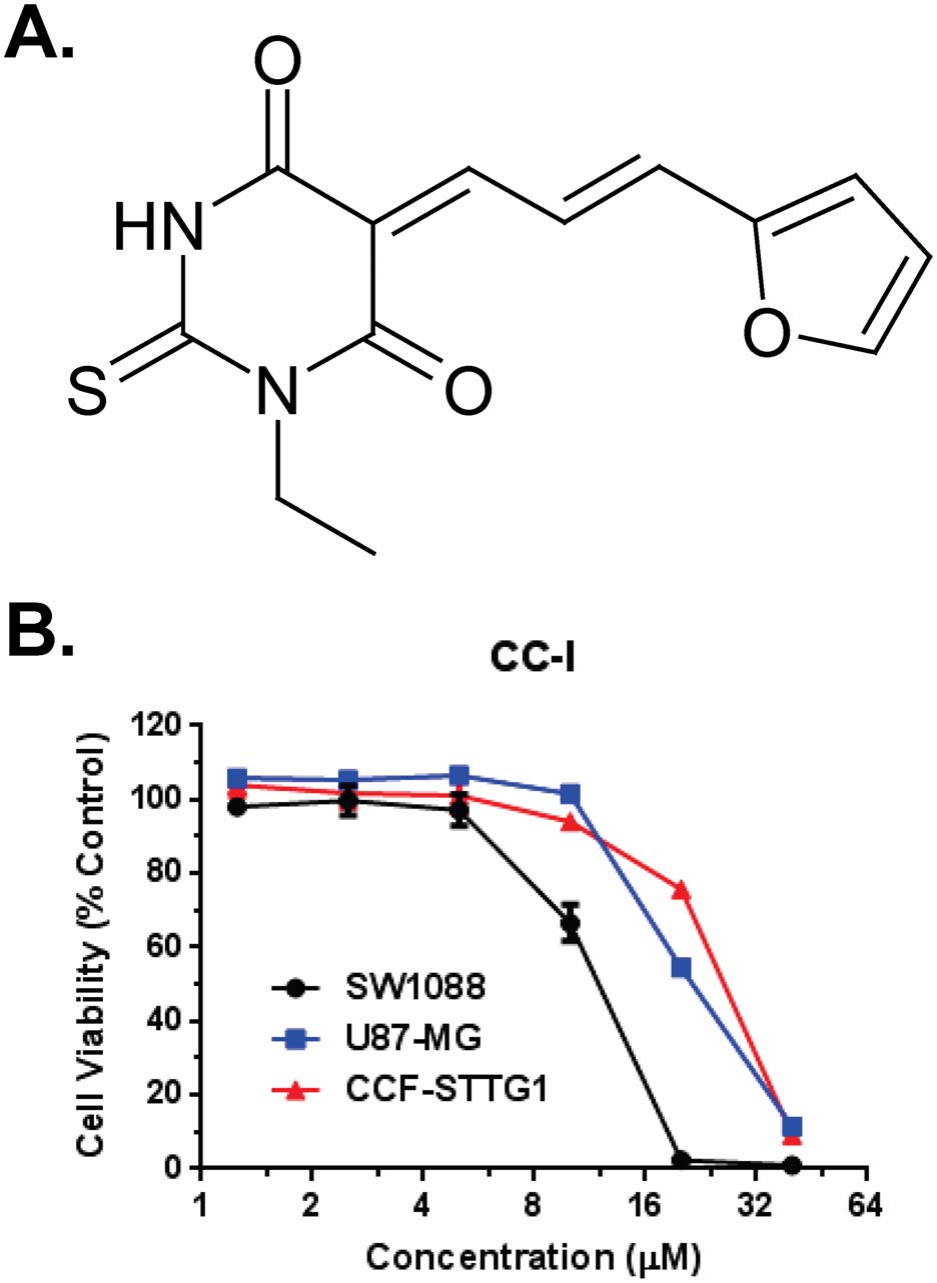
Chemical structure and cytotoxicity of CC-I in *in vitro*. (A) The structure of CC-I. (B) Cytotoxicity of CC-I in *in vitro*. Human astrocytoma cell lines were cultured with different doses of CC-I for 3 days and then the cytotoxicity was determined by SRB assay. The LC_50_ of CC-I to SW1088 cell lines (13.6 µM) are significantly different with the LC_50_ of CC-I to U87-MG and CCF-STTG1 cell lines (23.6 µM and 25.4 µM) (p<0.001).

Our screening approach identified a thiobarbituric acid analog and given the identification tag of chemotype compound–I (CC-I). The structure of CC-I is shown in **Figure 1A**. CC-I was cytotoxic to both the TMZ-resistant human astrocytoma cell lines CCF-STTG1 and to TMZ-sensitive SW1088 (**Figure 1B**). The LC_50_ of CC-I to SW1088, U87-MG and CCF-STTG1 cell lines is 13.6 µM, 23.6 µM and 25.4 µM respectively.

### Acute toxicity of CC-I in nude mice

Injections of CC-I once a week at 50 or 75 mg/kg body weight were lethal within 7 days. A once a week injection at 35 mg/kg body weight was tolerated. Therefore, we used approximately 70% of the tolerated dose (25 mg/kg body weight) of CC-I concentration for the *in vivo* tumor model study.

### Anti-tumor effect of CC-I in the subcutaneous mouse tumor model

**Figure 2:**
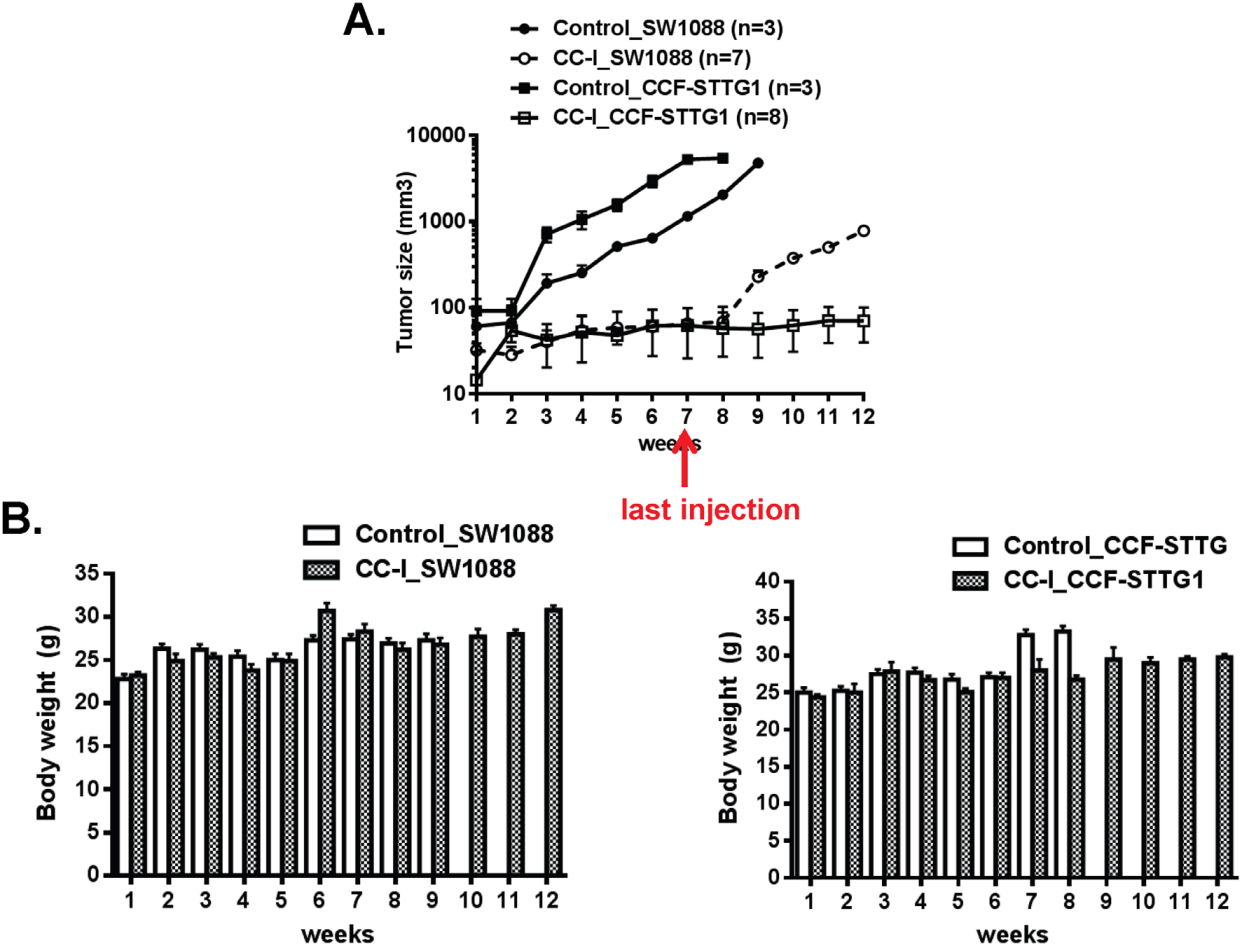
The anti-tumor effect of CC-I in a subcutaneous mouse tumor model. (A) Mice were implanted with ten million cells with the SW1088 or CCF-STTG1 cells. The starting tumor size for the CCF-STTG1 cells ranged from 80–100 mm^3^. The SW1088 cells grew more slowly so CC-I treatment was started when the tumors reached 30 mm^3^. CC-I was injected intraperitoneally at a concentration of 25 mg/kg body weight once a week for 7 weeks (n = 7∼10). The control group was given PBS in the same volume and regimen (n = 3–8). The tumor slowly reoccurred in the TMZ-sensitive SW1088 astrocytoma injected nude mice but did not reoccur in the TMZ resistant CCF-STTG1 injected nude mice when CC-I was discontinued (beyond 7 weeks). CC-I inhibited the tumor growth and was not lethal in any of the treatment groups. Some error bars are too small to be visible. (B) Mean body weight of mice is presented in grams. Some error bars are too small to be visible.

To establish the anti-tumor effect of CC-I on astrocytoma cells, we used the immunodeficient nude mouse subcutaneous tumor model injected with either TMZ sensitive SW1088 or TMZ resistant CCF-STTG1 cell lines. The mice with tumors from the CCF-STTG1 cell line showed no evidence of tumor progression following CC-I injections even after the injections ended (**Figure 2A**) whereas in the untreated control group the tumor volume dramatically increased over 7 weeks (p<0.0001). The tumors in mice from the SW1088 cell line also failed to progress during the injection period, but the tumor progressed when the CC-I injections were discontinued (**Figure 2A**). We did not take pictures of the tumors. We will consider taking pictures for upcoming experiments. The body weight for the control or CC-I treated mice did not decrease during course of the study (**Figure 2B**).

### Anti-tumor effect of CC-I in intracranial brain tumor model

**Figure 3:**
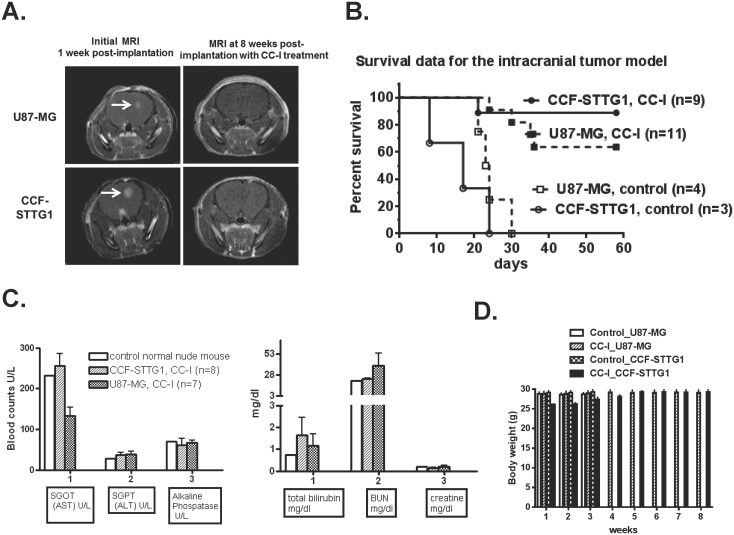
Anti-tumor effect of CC-I in an intracranial xenograft mouse model. (A) Representative MRI images taken with T1-weighted MRI contrast (7T MR imaging system) after intracranial tumor formation (one-three weeks post-implantation of astrocytoma cells) or after tumor formation followed by injection of CC-I (25 mg/kg body weight) for 7 weeks. CC-I completely inhibited tumor growth in both astrocytoma cell lines. (B) Kaplan-Meier survival graph of intracranial brain tumor mice after the administration of CC-I. CC-I extends the survival of the mice when compared to the untreated mice (n = 9 or 11) (p<0.0001). None of the mice which received PBS (control) survived after 30 days and median survival of all those animals was 20 days (n = 3 or 4). (C) Liver and kidney toxicity of CC-I. The liver and kidney toxicity (total bilirubin, blood urea nitrogen (BUN), creatine, aspartate aminotransferase (AST), alanine aminotransferase (ALT), and alkaline phosphatase) were determined using an automated chemistry analyzer machine (Roche Cobase MIRA) and kits manufactured by Thermo Electron. These data indicate no liver or kidney toxicity by CC-I in nude mice. Toxicity data displayed as means ± SEM. (D) Mean body weight of mice in grams.

After establishing the *in vivo* efficacy and safety of CC-I against both TMZ sensitive and resistant cell lines in the subcutaneous brain tumor model, we examined the intracranial xenograft brain tumor model. U87-MG or CCF-STTG1 astrocytoma cells were injected into the mouse brain and formed tumors (verified by MRI) ∼3 weeks post implantation (**Figure 3A**). None of the untreated control mice survived more than 30 days, and the median survival was 20 days. If the mice were being treated with CC-I, however 64% (7/11) of the U87-MG tumor bearing mice were still alive at 60 days and 89% (8/9) of the CCF-STTG1 tumor bearing mice were still live at 60 days (p<0.0001) (**Figure 3B**) and no tumor was visible on MRI (**Figure 3A**). Five mice in the U87-MG tumor group and six in the CCF-STTG1 tumor group receiving CC-I injections were alive 200 days after the tumor injection (137 days after the last CC-I injection). As with the systemic tumor model, there was no indication of liver or kidney toxicity from CC-I in intracranial xenograft mice (**Figure 3C**). The body weight of the animals did not decrease in the animals receiving CC-I (**Figure 3D**).

### Apoptosis of CC-I in the TMZ resistant astrocytoma cells

**Figure 4:**
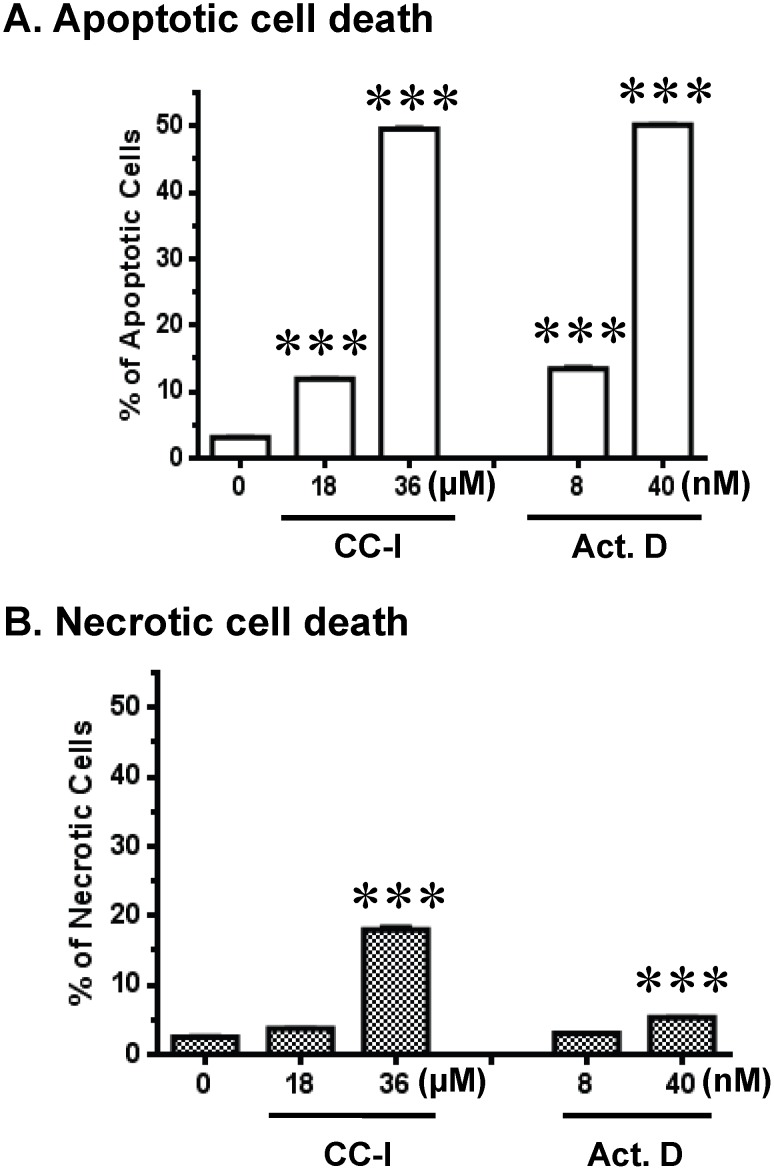
CC-I-induced cell death in CCF-STTG1 cells. Cell death was monitored with apoptotic and necrotic cell markers after 48 hours CC-I exposure in CCF-STTG1 cells. Cell death was determined with the recombinant annexin V conjugated to fluorescein, followed by flow cytometric analysis. Apoptotic cell death is shown in panel A. Panel B is necrotic cell death. Actinomycin D was used as a positive control to induce apoptotic cell death. The percentage of apoptotic cells following CC-I treatment was increased in a dose-dependent manner in CCF-STTG1 cells. There was not a pronounced dose dependent increase in necrotic cell death in the CCF-STTG1 cells until the higher concentration. Data assessed using Student *t* test and displayed as means ± SEM. Some error bars are too small to be visible. The symbols indicate a significant difference compared to the control. (***p<0.001).

Next we asked whether the cell death by CC-I to the TMZ resistant CCF-STTG1 astrocytoma cells is mediated through an apoptotic pathway. CC-I induced apoptosis in a dose dependent manner in CCF-STTG1 cell lines (**Figure 4A**). The amount of CCF-STTG1 apoptotic cell death at 36 µM was comparable to the positive control apoptosis inducer, actinomycin D. There is evidence of necrotic cell death in CCF-STTG1 following exposure to CC-I, but fewer cells were labeled and significance was not achieved until twice the concentration at which apoptosis was first observed (**Figure 4B**).

### Apoptosis gene array in CC-I treated TMZ resistant CCF-STTG1 cells

To determine which apoptotic pathway was activated by CC-I treatment, we performed gene expression profiles using targeted arrays for apoptosis. The Human Apoptosis Microarray revealed that tumor necrosis factor (TNF) pathway genes have the greatest changes in gene expression in the CC-I treated cells compared to the vehicle treated cells. CC-I (36 µM) increased TNF superfamily member 1, 2, 5, 6, and 9 as well as TNF receptor superfamily 5, 9, 10a from 30 to 700 fold. Among caspase pathway genes, only caspase 10 and caspase 14 were induced. The fold ratio of the altered genes is summarized in **Table 1**.

**Table 1:** Gene expression profile of human Apoptosis PCR Array in CC-I treated CCF-STTG1 cells.

*Gene Name (Gene Symbol)*	GenBankAccessionNumber	Description	Fold (Compare tocontrol)
*BAG-3/BIS (*BAG3*)*	NM_004281	BCL2-associated athanogene 3	up 8.2
*BCL-B/Boo (*BCL2L10*)*	NM_020396	BCL2-like 10 (apoptosis facilitator)	up 29.4
*BIP1/BP4 (*BIK*)*	NM_001197	BCL2-interacting killer (apoptosis-inducing)	up 9.9
*AIP1/API2 (*BIRC3*)*	NM_001165	Baculoviral IAP repeat-containing 3	up 10.4
*ILP-2/ILP2 (*BIRC8*)*	NM_033341	Baculoviral IAP repeat-containing 8	up 8.0
*ALPS2/FLICE2 (*CASP10*)*	NM_001230	Caspase 10, apoptosis-related cysteine peptidase	up 16.8
*MGC119078 (*CASP14*)*	NM_012114	Caspase 14, apoptosis-related cysteine peptidase	up 45.5
*Bp50/CDW40 (*CD40*)*	NM_001250	CD40 molecule, TNF receptor superfamily member 5	up 95.1
*CD154/CD40L (*CD40LG*)*	NM_000074	CD40 ligand (TNF superfamily, member 5, hyper-IgM syndrome)	up 52.2
*CIDE-A (*CIDEA*)*	NM_001279	Cell death-inducing DFFA-like effector a	up 27.3
*APT1LG1/CD178 (*FASLG*)*	NM_000639	Fas ligand (TNF superfamily, member 6)	up 33.3
*DP5/HARAKIRI (*HRK*)*	NM_003806	Harakiri, BCL2 interacting protein (contains only BH3 domain)	up 66.6
*LT/TNFB (*LTA*)*	NM_000595	Lymphotoxin alpha (TNF superfamily, member 1)	up 110.6
*ASC/CARD5 (*PYCARD*)*	NM_013258	PYD and CARD domain containing	up 6.3
*DIF/TNF-alpha (*TNF*)*	NM_000594	Tumor necrosis factor (TNF superfamily, member 2)	up 703.4
*APO2/CD261 (*TNFRSF10A*)*	NM_003844	Tumor necrosis factor receptor superfamily, member 10a	up 22.8
*S152/T14 (*CD27*)*	NM_001242	CD27 molecule	up 8.1
*4–1BB/CD137 (*TNFRSF9*)*	NM_001561	Tumor necrosis factor receptor superfamily, member 9	up 302.6
*CD27L/CD27LG (*CD70*)*	NM_001252	CD70 molecule	up 38.2
*CD153/CD30L (*TNFSF8*)*	NM_001244	Tumor necrosis factor (ligand) superfamily, member 8	up 22.3

### Effect of CC-I on the cell cycle of TMZ resistant astrocytoma cells

**Figure 5:**
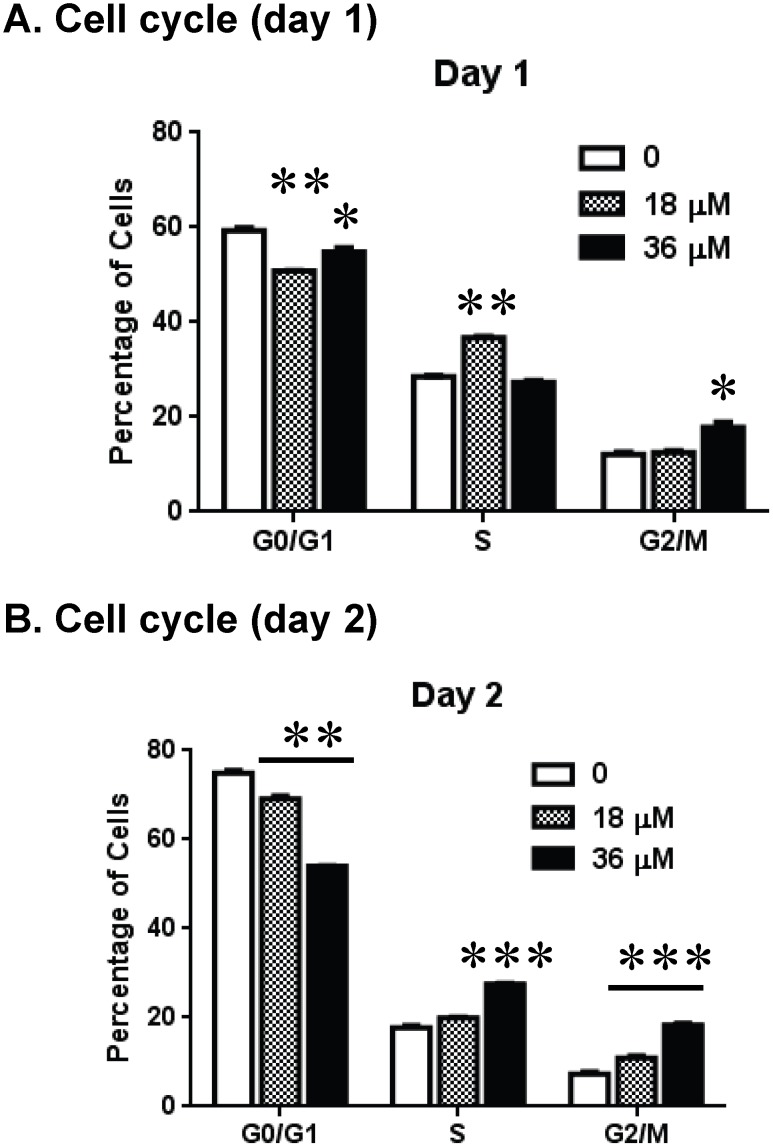
CC-I-induced cell cycle arrest in CCF-STTG1 cells. The CCF-STTG1 cells were treated with 18 or 36 µM of CC-I for 24 or 48 hours. The cells were stained with propidium iodide and then analyzed for cell cycle distribution using a FACScan analyzer. CC-I treatment significantly increased the S and G2/M cell population, but decreased in G0/G1 phase. The symbols indicate a significant difference compared to the control. (*p<0.05; **p<0.01; ***p<0.001).

To better understand the cytotoxic effect of CC-I, we performed a cell cycle analysis in CCF-STTG1 cells after CC-I treatment**.** CC-I treatment of CCF-STTG1 cells resulted in a significant decrease in the G0/G1 phase, and an increase in the S and G2/M phase compared to untreated cells (**Figure 5A & B** **)**.

### Topoisomerase IIα inhibition by CC-I

**Figure 6:**
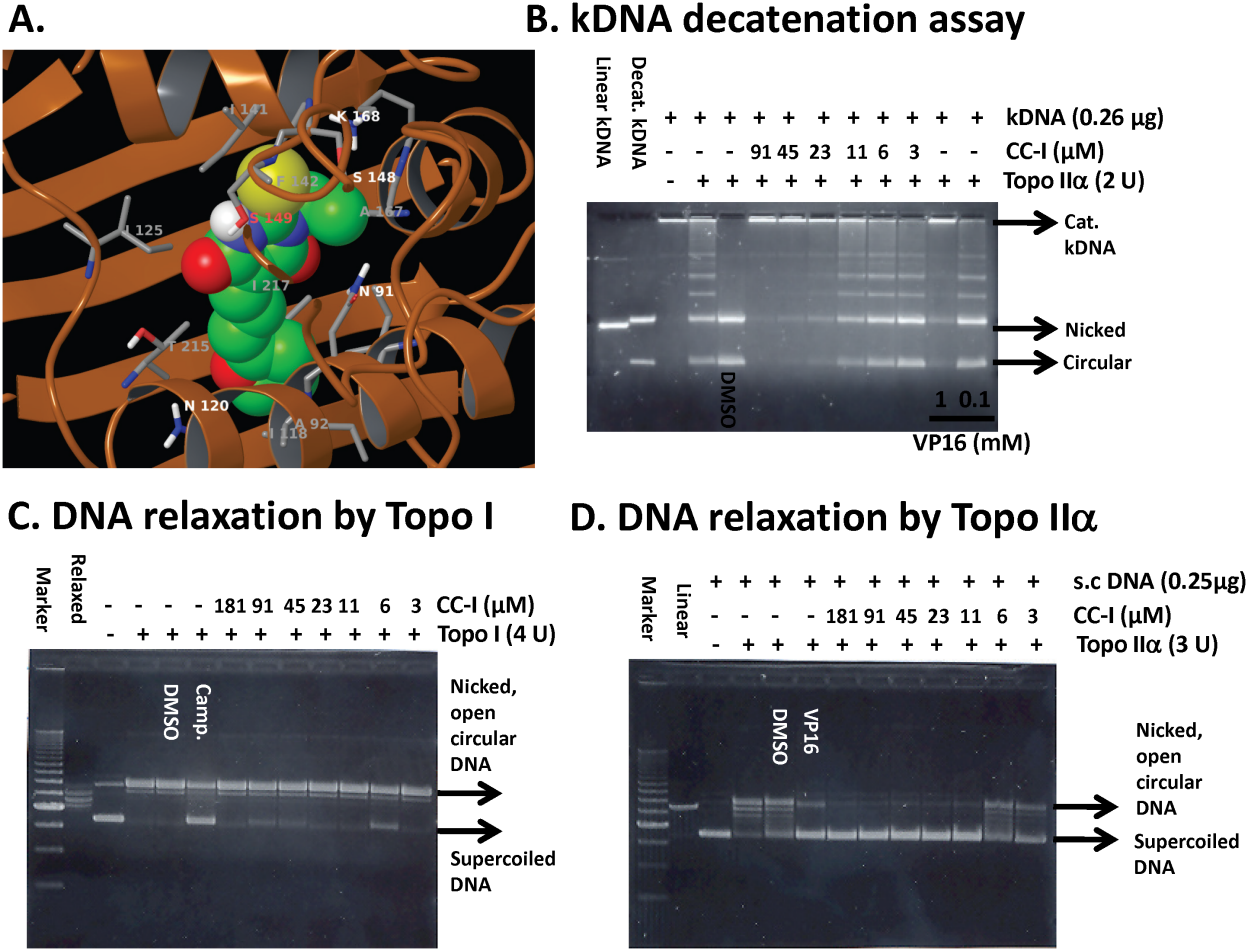
Topoisomerase IIα inhibition by CC-I. (A) Structure of CC-I docked into topoisomerase IIα (pdb code 1ZXM). Topoisomerase is shown as the brown-colored ribbon with residues on the binding site. Carbon atoms of CC-I are colored green, while those of topoisomerase is colored gray. Other atoms are colored according to atom types, i.e., nitrogen-blue, oxygen-red, sulfur-yellow, and polar hydrogen white. Non-polar hydrogen atoms are not shown. (B) The CC-I concentration-dependent inhibition of human topoisomerase IIα-mediated kDNA decatenation. All experiments were carried out according to instructions from the Topogen kit (Port Orange, FL). Reactions contained 4U of enzyme, 0.26 µg of DNA substrate, and different concentrations of the CC-I dissolved in DMSO (0.5% final concentration (v/v)). Different topological forms exhibited different mobility as indicated. Linear, linear kDNA; Decat., decatenated kDNA; Nicked, nicked decatenated kDNA; circular, circular decatenated kDNA; kDNA, kinetoplast DNA. VP16 was used as a positive control. (C) CC-I did not inhibit topo-I mediated supercoiled pHOT1 DNA relaxation. The procedures are described in method section. Camptothecin (camp.) was used as a positive control. (D) CC-I dose dependently inhibited topoisomerase IIα-mediated supercoiled pHOT1 DNA relaxation. VP16 was used as a positive control. s.c. DNA, super-coiled DNA.

We determined whether CC-I can bind human topoisomerase IIα in a molecular modeling study. The molecular modeling data between human topoisomerase IIα and CC-I suggested that CC-I fits into the cavity of human topoisomerase IIα where it could function as an inhibitor (**Figure 6A**). Therefore, we performed DNA relaxation and kDNA decatenation assays to determine the ability of CC-I to inhibit topoisomerase IIα enzyme activity. CC-I inhibited topoisomerase IIα activity in a dose dependent manner. At concentrations greater than 23 µM, CC-I inhibited topoisomerase IIα catalyzed kDNA decatenation (**Figure 6B**). Etoposide (VP16), a known topoisomerase II poison, inhibited topoisomerase IIα at 1 mM but not at 0.1 mM concentration (**Figure 6B**). Next, we determined whether CC-I is a specific inhibitor of topoisomerase IIα using a supercoiled DNA relaxation assay. CC-I did not enhance topoisomerase I-mediated relaxation of supercoiled pHOT1 DNA (**Figure 6C**). Camptothecin, a topoisomerase I inhibitor, was used as a positive control for the assay and showed the expected inhibition of topoisomerase I mediated DNA relaxation. In contrast, CC-I exhibited a strong inhibitory effect on topoisomerase IIα-mediated relaxation of supercoiled pHOT1 DNA (**Figure 6D**). The effective concentration of CC-I on topoisomerase IIα mediated DNA relaxation was first seen at 11 µM.

### Comparison of cytotoxicity between CC-I and topoisomerase inhibitors on the astrocytoma cells

**Figure 7:**
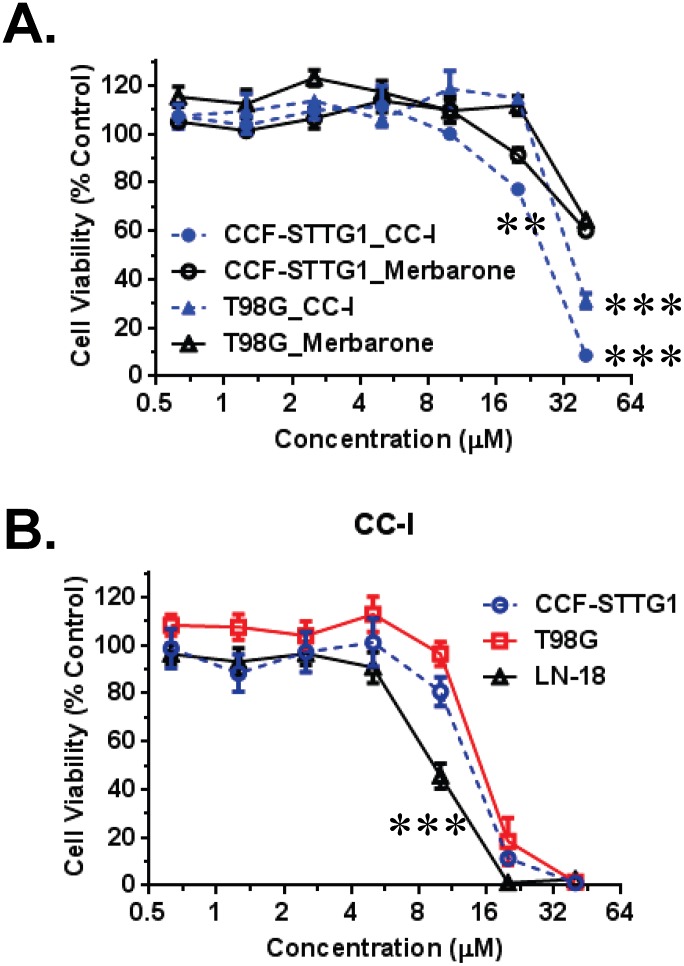
Cytotoxicity of CC-I, merbarone, and combination of CC-I and TMZ on the astrocytoma cells. (A) TMZ-resistant human CCF-STTG1 and T98G cell lines were cultured for 3 days with CC-I and other similar structure topoisomerase II inhibitor (merbarone) followed by cytotoxicity measurement by SRB assay. CC-I showed greater toxicity than merbarone on the astrocytomas. The symbols indicate a significant difference between the merbarone treated and CC-I treated groups (**p<0.01; ***p<0.001). (B) The MGMT methylated (T98G, CCF-STTG1) or un-methylated (LN-18) astrocytoma cell lines were cultured for 3 days with CC-I and determined cytotoxicity by SRB assay. T98G cells have methylated MGMT promoter, but show weak MGMT expression. CC-I is more cytotoxic to LN-18 cells which has un-methylated MGMT promoter and MGMT expression. The symbol (***) indicates the most difference between the cells (p<0.001).

We compared the relative toxicity of structurally similar topoisomerase inhibitors using TMZ resistant CCF-STTG1 and T98G cells (**Figure 7A**). The LC_50_ of CC-I for CCF-STTG1 and T98G astrocytomas was approximately 22.5 and 29.1 µM. The LC_50_ concentration for CC-I is significantly lower than that found for merbarone (LC_50_: >40 µM, p<0.01). We observed similar relative toxicity of these compounds on SW1088 and U87-MG cell lines.

### Cytotoxicity of CC-I on the MGMT promoter methylated and unmethylated GBM cells

We determined the effect of CC-I using several GBM cell lines that have different MGMT promoter methylation status and MGMT protein expression levels. The LN-18 cell line, which has unmethylated MGMT promoter and MGMT protein expression [26], [34], is more sensitive to CC-I than CCF-STTG1 or T98G cells (LC_50_: 9.03 µM, 14.8 µM, and 13.5 µM respectively; p<0.05) (**Figure 7B**). The latter cells have methylated MGMT promoter [26].

### Combination effect of CC-I and TMZ on the TMZ resistant astrocytoma cell line

**Figure 8:**
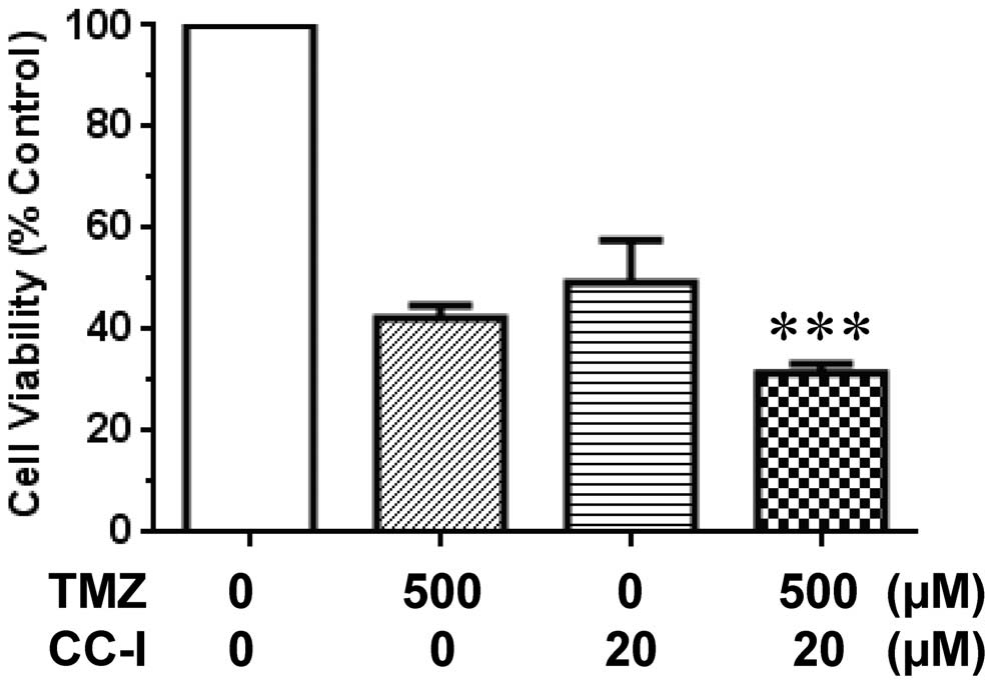
Combination effect of CC-I and TMZ on the T98G astrocytoma cells. T98G cells were cultured for 3 days with CC-I and TMZ, and cytotoxicity was evaluated by SRB assay. Both CC-I and TMZ treatment on the T98G cells showed much more cytotoxic effect than either single treatment. The symbol (***) indicates a significant difference between the control and single treatment groups (p<0.001).

To test whether CC-I can enhance cytotoxicity of TMZ in astrocytoma cell lines, we determined effect of combination of both drugs (CC-I & TMZ) on the survival of CC-I resistant T98G cell lines. Survival of cells was evaluated following treatment with concentrations of CC-I and TMZ around their respective the LC_50._ There was an additive effect of both drugs. Cell survival which was significantly (p<0.001) reduced in the combined therapy group compared to single treatment in T98G cells after 3 days exposure (**Figure 8**).

## Discussion

The present study investigated the development of anti-tumor compounds for TMZ resistant cancer cell lines. Using TMZ resistant cancer cell lines, we identified a lead compound CC-I which is an analog of thiobarbituric acid. The results of the *in vivo* study demonstrate that CC-I is a safe and effective anti-tumor compound against astrocytoma cell lines, including those shown to be resistant to chemotherapy and radiation. CC-I induced apoptosis and cell cycle arrest in astrocytoma cells. Because of its structural similarity to topoisomerase inhibitors, we examined CC-I for topoisomerase inhibition and found it selective for topoisomerase IIα. The cytotoxicity of CC-I is greater than other compounds of similar structure.

We have previously reported that human neuroblastoma cells and human astrocytoma cells lines expressing commonly occurring polymorphisms in the HFE gene were resistant to chemotherapy and radiation [26]. The CCF-STTG1 astrocytoma cell lines that carry the HFE C282Y gene variant were even more resistant to TMZ than T98G or U343-MG cell lines, which are considered standards for TMZ resistance [26], [35], [36]. The CCF-STTG1 cells are also resistant to geldanamycin, its derivatives, and radiation [26] and less sensitive to merbarone; a compound chemotypically similar to our CC-I compound that reached Phase II clinical trials. Our approach using TMZ resistant astrocytoma cells was successful and identified a lead therapeutic agent, CC-I, with strong cytotoxicity to tumors, prevention of tumor recurrence, and an acceptable safety profile in *in vivo*. Tumors did not return in 45–66% (depending on cell line) of the mice for 151 days after the last injection and the mice were still alive at 200 days of age when the study was terminated.

CC-I belongs to the thiobarbituric acid family. Various barbituric acid derivatives have been studied as anti-inflammatory and anti-cancer compounds [37]–[39]. Thiobarbituric acid derivatives also have been studied as anti-tumor agents, uridine phosphorylase inhibitors, HIV-1 integrase inhibitors, and hepatitis C virus polymerase inhibitors [40]–[43]. An example of thiobarbituric acid derivative evaluated as a treatment for brain cancer is merbarone [5-(N-phenylcarboxamido)-2-thiobarbituric acid] which has a similar structure to CC-I. Merbarone is a non-sedating derivative of thiobarbituric acid and induces single strand breaks in DNA apparently without binding to DNA [44], [45]. CC-I also shares structural similarity with ICRF-193 which is a bisdioxopiperazine derivative compound. It has been reported that merbarone and ICRF-193 inhibit topoisomerase [46]. The present study demonstrated that CC-I also inhibits topoisomerase activity within a similar concentration range to merbarone but CC-I is more cytotoxic to the TMZ resistant CCF-STTG1 astrocytoma cell lines than these two compounds. The reason for the differences in cytotoxicity may be due to a structural difference between CC-I which has diene motif linking the barbiturate C5 position with the terminal aromatic ring rather than a shorter amide linker as in merbarone. There is also a structure difference in the functional residue at N1 position; CC-I compound has N-ethyl group, but merbarone has a NH residue.

CC-I exposure resulted in S and G2/M arrest in CCF-STTG1 astrocytoma cell line. This observation is consistent with a number of anti-tumor agents such as 9-methoxycamptothecin, topoisomerase II poisons (doxorubicin, etoposide) [47], [48]. For example, 9-methoxycamptothecin induced apoptosis through TNF and Fas/FasL pathway, oxidative stress, and G2/M cell cycle arrest in multiple cancer cell lines [47]. Camptothecin, a topoisomerase I poison, also triggers S and G2/M arrest in cancer cell lines [49]. Our PCR array data indicate that CC-I induces cell death through TNF signaling pathway and the Annexin V data indicate cells die via apoptosis. Therefore our present cell cycle analysis study indicates that CC-I has a similar impact on cell cycle and subsequent apoptosis as many anti-cancer compounds.

CC-I was identified by screening against TMZ resistant astrocytoma cells. However, CC-I was also toxic to TMZ sensitive astrocytoma cells (SW1088, U87-MG). In vivo, CC-I showed greater efficacy against TMZ resistant CCF-STTG1 subcutaneous and intracranial tumors than TMZ sensitive astrocytoma cells (**Figure 2A** **&** **3B**). MGMT methylation status influenced CC-I cytotoxicity, but CC-I has a lower LC_50_ than regardless of methylation status compared to TMZ [26]. This finding is important because there is a correlation between MGMT promoter methylation and GBM patient survival [50]. Because of the relative differences in effect based on methylation status (and HFE genotype) we investigate CC-I in combination with TMZ and found the addition of CC-I improves TMZ efficacy in TMZ resistant astrocytoma cell lines. These findings are consistent with several studies reporting a combination effect with an anti-tumor compound and TMZ in TMZ resistant astrocytoma cell lines [51], [52]. The data suggest that CC-I could be considered an adjuvant therapy with TMZ. There are many limitations in translating studies, such as ours, that find compounds that show efficacy in animal models to clinical application. Nonetheless, the results of the initial analyses of CC-I warrant further investigation.

In conclusion, we identified an anti-tumor compound for TMZ resistant and sensitive astrocytomas with strong *in vivo* efficacy and safety profiles in mouse tumor models. The cytotoxicity of CC-I is mediated by apoptosis, cell cycle arrest at S and G2/M phase. CC-I has a similar biological profile to other topoisomerase inhibitors but it is smaller and shows effects in orthotopic models, therefore we believe it has more attractive properties than most other topoisomerase inhibitors that allows it access the brain.
